# A mobile school-based HCT service – is it youth friendly?

**DOI:** 10.1080/17290376.2016.1222597

**Published:** 2016-08-31

**Authors:** Estelle Lawrence, Patricia Struthers, Geert Van Hove

**Affiliations:** ^a^ PhD student, School of Public Health, University of the Western Cape, Cape Town, South Africa; ^b^ PhD, Associate Professor at School of Public Health, University of the Western Cape, Cape Town, South Africa; ^c^ PhD, Professor at Department of Special Education, Ghent University, Ghent, Belgium

**Keywords:** HIV/AIDS, HIV Counselling and Testing, youth, students, school, youth friendly, VIH/SIDA, Aide Psychosociale et Test de Dépistage VIH (ADV), jeunes, étudiants, école, adapté aux besoins des jeunes

## Abstract

Background: Despite an increase in HIV Counselling and Testing (HCT), few young people have been tested. It has been suggested that they do not test because formal health services (where HCT is provided) are often not youth friendly. The World Health Organisation describes a youth-friendly health service (YFHS) as one which is accessible, equitable, acceptable, appropriate, and effective. A mobile school-based model has been implemented by a non-governmental organisation in Cape Town in an attempt to make HCT more youth friendly and accessible to young people. The objective of this study was to explore whether this mobile school-based HCT service is youth friendly. Methods: The study was descriptive, using three qualitative data collection methods: observation of the HCT site at two secondary schools; interviews with six service providers; and direct observation of 21 HCT counselling sessions. Key Results: The mobile school-based HCT service fulfilled some of the criteria for being a YFHS. The service was equitable in that all students, irrespective of race, gender, age, or socio-economic status, were free to use the service. It was accessible in terms of location and cost, but students were not well informed to make decisions about using the service. The service was acceptable in that confidentiality was guaranteed and the service providers were friendly and non-judgemental, but it was not considered acceptable in that there was limited privacy. The service was appropriate in that HCT is recommended as an intervention for decreasing the transmission of HIV, based on evidence and expert opinion; however, in this case, HCT was provided as a stand-alone service rather than part of a full package of services. Moreover, studies have suggested that young people want to know their HIV status. The service was ineffective in that it identified students who are HIV positive; however, these students were not assisted to access care. Conclusion: Providing HCT in the school setting may make HCT more accessible for students, but it needs to be provided in an equitable, accessible, acceptable, and effective way.

## Résumé

Contexte: Malgré l’augmentation en Aide Psychosociale et Test de Dépistage VIH (ADV), peu de jeunes ont subi de test. On a suggéré qu’ils ne subissent pas de tests parce que le service officiel de santé (où l’ADV est apporté) n’est pas toujours adapté aux besoins des jeunes. L’Organisation Mondiale de la Santé qualifie un service de santé adapté aux besoins des jeunes (SSAJ) comme étant accessible, équitable, acceptable, approprié et efficace. Un modèle mobile basé à l’école a été mis en application par une organisation non gouvernementale à Cape Town pour essayer de rendre l’ADV plus accessible aux jeunes et mieux adapté à leurs besoins. Le but de cette étude était d’examiner si le service ATV mobile basé à l’école est adapté aux besoins des jeunes. Méthodes: L’étude a été descriptive utilisant trois méthodes de collecte de données qualitatives: observation du lieu de l’ATV dans deux écoles secondaires ; interviews avec six fournisseurs de service et observation en direct de 21 séances d’aide psychosociale ADV. Résultats clés: Le service ADV mobile basé à l’école a répondu à certain des critères pour être un SSAJ. Le service était équitable en ce que tous les étudiants, sans distinction de race, sexe, âge ou position socio-économique étaient libres d’utiliser le service. Il était accessible en termes d’emplacement et de frais, mais les étudiants n’étaient pas bien informés pour pouvoir prendre la décision d’utiliser le service. Le service était acceptable en ce que la confidentialité était garantie et les fournisseurs de service étaient sympathiques et neutres, mais il était inacceptable en ce qu’il n’était pas suffisamment privé. Le service était approprié en ce que l’ADV est recommandé comme intervention pour réduire la transmission du VIH, basé sur les preuves et d’après les experts, cependant dans ce cas, l’ADV a été apporté comme un service autonome plutôt qu’une partie d’un ensemble complet de services. En plus, des études ont suggéré que les jeunes veulent savoir leur position VIH. Le service était ineffectif en ce qu’il a identifié les étudiants qui sont séropositifs, cependant ces étudiants n’ont pas eu d’aide pour avoir accès aux soins. Conclusion: L’apport de l’ADV dans un cadre scolaire pourrait rendre l’ADV plus accessible aux étudiants, mais il doit être apporté de façon équitable, accessible, acceptable et efficace.

## Introduction

The HIV epidemic is a major public health problem among youth in South Africa. In the 2012 South African National HIV Prevalence, Incidence, Behaviour, and Communication Survey, levels of HIV infection among 15- to 24-year olds (despite a decrease) were reported as still being high, with 5.6% and 17.4% prevalence among young women aged 15–19 and 20–24 years old, respectively, and 0.7% and 5.1% prevalence among young men aged 15–19 and 20–24 years old, respectively (Shisana, Rehle, Simbayi, Zuma, Jooste, Zungu, *et al*. 2014).

In accordance with Joint United Nations Programme on HIV and AIDS (UNAIDS) (1997) recommendations, one of the ways that the South African government had tried to address this problem was to include HIV testing in the National HIV and AIDS and Sexually Transmitted Infections (STIs) Strategic Plan for 2007–2011 (South African National AIDS Council 2007), with a specific focus on young people. As part of this strategy, the National Department of Health’s HIV and AIDS and STI Strategic Plan for 2012–2016 (South African National AIDS Council 2011) includes HIV testing in schools; however, it acknowledges that it is not part of the South African Department of Basic Education’s draft HIV and AIDS Strategic Plan for 2012–2016 (South African Department of Basic Education 2012) and that policies guiding HIV testing in schools still need to be developed.

However, since the mid-1990s, non-governmental organisations (NGOs) in Cape Town have been providing an HIV Counselling and Testing (HCT) service at secondary schools where school governing bodies have given permission. The HCT service provided by one of these NGOs is the focus of this study.

This particular NGO contacts schools, and those schools that decide to utilise their service are visited to make preparations for HCT to take place. On the testing day, students are called class by class to the school hall (or a similar space). Here the HCT procedure is explained, and then those who decide to be tested stay behind, whilst the rest return to class. The remaining students each receive individual pretest counselling, followed by a finger-prick, rapid HIV test. Fifteen minutes after they have been tested, they are given their results in conjunction with post-test counselling. Students who test HIV positive are referred to a health facility for further management. Testing at each school usually lasts a number of days, depending on the student numbers at the school, and the demand for HCT.^1^

Little has been published about this school-based model of HCT provision. A mobile school-based service in Uganda run by the Kitovu Mission Hospital claimed successful provision of HCT in a school setting (as illustrated in Boswell & Baggaley 2002). In South Africa, Pfaff and de Beer (2011) describe a school-based HCT strategy, aimed at young people in KwaZulu-Natal; and Madiba and Mokgatle (2015) found a high acceptability of HCT at schools in Gauteng and North West provinces.

However, these programmes do not replicate any model, and need to be evaluated. In this study, the school-based HCT service is evaluated using the WHO’s (2002) criteria for an adolescent-friendly^2^ health service. The WHO describes an adolescent-friendly health service as being ‘accessible, equitable, acceptable, appropriate and effective’. They recommend that health services for adolescents should have policies, procedures, facilities, and staff that are adolescent friendly, and should include youth and community involvement (WHO 2002).

## Methodology

This evaluation was exploratory and descriptive, and consisted of an observation of the HCT sites, service provider interviews, and direct observation of the HCT counselling process. The study population was the mobile school-based HCT service provider team, which comprised the project manager, two nurses, and four counsellors. All members of the team except one counsellor (who declined) took part in the interviews. The observation of the HCT sites took place at two high schools (School A and School B), which were selected using convenience sampling. They were the only schools at which the NGO conducted tests during the time that data were collected. Data were collected on six non-consecutive days over a six-week period. The data were not collected sequentially (i.e. first the interviews, then the observations), but occurred at times that were convenient for the researcher and for the service providers.

All members of the team were observed at the HCT site, and all four counsellors’ sessions were observed and audio-recorded. In total, 21 counselling sessions (14 pretest and 7 post-test sessions) were convenience sampled and directly observed at the two schools where the site visits took place.

Questions to guide the interview and the observation of the HCT site were taken from an amended assessment tool, ‘Clinic Assessment of Youth Friendly Services’ (Senderowitz, Solter & Hainsworth 2002); the tool was originally developed to be used by project managers to assess clinic-based services where young people are seen at a facility at which adults are also treated, and where services other than HCT are available. Because this study was intended for evaluating HCT at *schools* specifically, the tool was amended to make it more appropriate for a mobile school-based service (see Appendix 1 for amendments). The amendments were minimal; so reliability and validity tests were not conducted on the amended tool. UNAIDS Tool 4 (UNAIDS 2000), which is designed to assess the standards of HIV counselling by monitoring the process, content, and quality of pre- and post-test HIV counselling sessions, was used during the direct observation of the HCT counselling process.

The HCT service providers agreed to be part of the study, and the research proposal was discussed with them prior to ethical approval being obtained from the University of the Western Cape Senate Ethics Committee. Each service provider gave written informed consent to be interviewed, which included permission to observe and audio-record counselling sessions.

A directed content analysis approach was used to analyse the interviews; this involved developing predetermined coding categories which then directed the analysis of the data. The headings and questions in the amended tool ‘Clinic Assessment of Youth Friendly Services’ (Senderowitz *et al*. 2002) and the UNAIDS Tool 4 (UNAIDS 2000) were used as coding categories. To validate the analysis, coding was also done by an independent researcher. The notes and diagrams made during the observation of the HCT sites were expanded into a narrative describing the HCT site environment and process at the two schools. The transcripts of the audio-recordings of the HCT counselling sessions were read, and the content and quality of the counselling were scored using the UNAIDS Tool 4 checklist (UNAIDS 2000). These scores were then checked to see if they coincided with the scores that had been given on the day of observation. The notes that had been made during the observation of the sessions were used to add to the description of the counselling sessions. In addition, when the HCT sites were visited for non-research purposes (e.g. to arrange interviews) and the service providers were informally observed, they did not change their behaviour.

## Findings

### Findings from the service provider interviews

#### Profile of service providers

In Table 1, the gender, age, and job title of the service providers are presented. Except for the project manager, all providers were female. The youngest counsellor was 34 years and the oldest counsellor, 71 years.

**Table 1.** Profile of service providers.

Title	Gender	Age
Project Manager	Male	52
Professional Nurse	Female	67
	Female	63
Counsellor	Female	71
	Female	34
	Female	48

#### Service providers’ work experience and training

The project manager was a qualified pastor and, therefore, had had pastoral counselling experience. Both nurses had worked in primary healthcare clinics for more than 30 years where they had had minimal contact with young people. They acquired experience in HIV and AIDS, HIV testing, and treatment of STIs when working in the clinics. One of the nurses had been working with the NGO for one year, and the other for six years. All the counsellors had minimal experience working with young people. Except for one of the nurses who had done a course on Teenage Sexuality in the 1980s, none of the providers had received any youth-specific training. All the providers had had training regarding HIV, AIDS, and antiretroviral therapy as well as counselling skills specific to HCT.

#### Policies and guidelines

No guidelines or policies existed for providing HCT to students at the schools in the sample, and the NGO had no written policies or guidelines of their own. The project manager said he followed the legal guidelines for general HIV testing in South Africa (South African Department of Health 2010).

#### Parental consent

According to South African Law, parental consent for HIV testing is not required for students over the age of 12 years (Republic of South Africa 2006). The project manager encouraged the school to inform parents that testing would take place, but apparently this was only done by School B. All students gave consent during pretest counselling.

#### Providing students with information about HCT

The project manager explained that before the HCT takes place at a school, he meets with the principal and the contact teacher. He assumes that the school informs the students about the testing event. However, during focus group discussions with the students, some mentioned that they were not told beforehand that testing would take place.

On the day of testing, the whole class is called down to the testing area. As a group, they are educated by the project manager about the prevalence of HIV, the importance of testing, how the testing process will take place, and that confidentiality is guaranteed. The project manager notes:

… so that was very important … that you get all the students first, get them down, explain to them, allay their fears, because they were fearful about this whole thing, you know. They were fearful about HIV testing, so you had to make sure … for example a lot of them had a phobia for needles so you had to explain to them, listen, this is finger pricks, it’s not long needles for example, otherwise they wouldn’t test …

… they felt more eager to come and test after I would explain to them for example that the law guarantees confidentiality and no information must be or should be given personally about them to the school or to the principal or to anybody whatsoever … I think to a large extent that really made them feel at ease and they would come and test …

#### When HCT takes place

According to the service providers, testing takes place during school hours, from 0900 to1500 hours. The providers explained that every effort is made to respect curriculum time and not to disrupt school functioning. Times of testing are, therefore, usually arranged with the Life Orientation (LO)^3^ teacher (who is the contact person at each school), and testing generally takes place during the LO period. Testing is not normally scheduled during interval, but students do sometimes drop in during this time.

#### Privacy

The service providers explained that testing takes place in the school hall at most schools. If the school does not have a hall, then an alternative big space, such as the library, is used, or counsellors set up in separate empty classrooms. Previously, tents had been set up in the hall; but about six months prior to the interviews, the tents had been broken, so instead they placed tables far apart in the hall to try to ensure auditory privacy. At some schools, the school provided screens to create cubicles in an attempt to provide some visual and auditory privacy (as School B had done). Service providers voiced their concerns that this set-up was not ideal, as they felt that both visual and auditory privacy are important to ensure confidentiality. A counsellor noted:

Without the tents or the cubicles, the other children who’s waiting on the other side, they can overhear everything that’s said.

For this reason, the service providers tried to provide some sort of privacy for students receiving their results, by setting the post-test counselling table behind a makeshift screen or curtain. One counsellor felt that results should be given separately from the other students, as they could often work out from the time it takes to give results whether the result is positive or negative.

The project manager stated that he visits the site before HCT takes place to check that they have a space that is suitable for testing. He explained:

I would physically look at what space is available … and then only after I’m satisfied that the process or the HIV AIDS counselling and testing can be done in a safe environment, then I would say, okay, it’s fine.

Despite this, he mentioned an episode at one of the schools (where tents were used in a hall) where complaints were made because students waiting to be tested had overheard a counselling session taking place in one of the tents.

#### Confidentiality

No written procedures exist for protecting the students’ confidentiality; however, the team tries to ensure confidentiality as they have been taught this during their training. The project manager follows the legal guidelines regarding confidentiality in HIV testing. He noted:

That’s very important because I have often refused to test at some of the schools if confidentiality cannot be guaranteed.

All forms and registers are handled only by the professional nurses and the project manager, and are stored in a locked cupboard at their offices. No paper trail is left at the school. There is no registration process, except at School B, where all students who come down to the HCT site sign a register, irrespective of whether they are tested or not.

#### Counselling difficulties

Counsellors complained about the new ACTS^4^ method of counselling, which does not include taking a sexual risk history during pretest counselling. The ACTS form (see Appendix 2) does not include the question ‘Are you sexually active?’ One of the counsellors, however, did add this question during pretest counselling, so that she could address issues regarding safer sex and pregnancy prevention during post-test counselling.

One counsellor expressed her unhappiness with the fact that students seemed not to be interested in discussing prevention plans, and therefore she often omitted it from the counselling process.

The post-test counselling, I make it shorter because the children just want to know their results … They will … stand like this … I don’t want them to stand; they must sit so that even if the client is tested negative, I have to talk about the negative, or the positive lifestyles, you see … they’re just in a hurry to hear their results. (Counsellor)

Counsellors felt that not enough time was allowed for counselling.

Counsellor: When you are in the clinics … you see 12 [clients] but in the schools you cannot do 12 … they [the Department of Health] want the stats [statistics]. They run for the stats. And to me it’s not about stats; it’s about the quality of the job that you are doing.

Interviewer: So how many students do you see generally in a day?

Counsellor: In a day, you see more than 20. Serious, we see more than 20.

…

Counsellor: You’re being rushed the whole time while you’re there. You need to make up your stats, you can’t still think of whatever. Because sometimes there’s other problems that the kids have … they don’t just come sit there for HIV, they come sit there to tell you something else, something more personal. And now you get somebody rushing you on that side, so you can’t really listen …

#### Health needs other than HCT

Counsellors reported that students sometimes asked for treatment for STIs and pregnancy testing. The nurses then referred them to the local health facility. One counsellor described her experience of accompanying a student to a clinic for treatment of an STI.

Counsellor: There was the girl with that STI but she was scared to go to the clinic, and she actually showed me. She pulled down her pants and she showed me, and then [the project manager] said it’s okay, I can take her to the clinic.

Interviewer: Why do you think she was scared to go to the clinic, any idea? Did she tell you?

Counsellor: She said … that the people in the area might see her … and she had blisters, hey, but the blisters were, like, far already, so I told her let’s go into another area … and I told [the project manager] I can’t let this child go alone because she was only 15, so I went with her and we went to the clinic.

The NGO’s policy on providing condoms was not clear, but it appeared as though they had handed out condoms to students in the past. However, on the days that the HCT site visit was done, the team did not have condoms.

#### Psychosocial needs

Service providers said that students often raised psychosocial problems when they were counselled for HCT, such as physical and emotional abuse and bullying. The project manager reported:

HIV testing and counselling goes way beyond HIV testing at the school, because … you find yourself dealing with so many issues of the student: abuse, rape, incest, physical abuse, emotional abuse in the home environment.

The project manager voiced concerns that the abridged counselling, using the ACTS form, limited opportunities for students to bring up these issues. He noted:

They used to ask on the questionnaire [UNAIDS model of HCT] quite a few in-depth questions. For example if you’re found to be HIV positive, you know, ‘What will you do?’ ‘Who will you confide in, and how about your sexual partner?’ But those questions are not part of the current questionnaire [ACTS model] that we’re using, which means it has really, sort of, minimised the time that you spend with a student.

#### Referral and follow-up of students

Students who tested positive are counselled by an NGO counsellor and given a standardised referral letter by the nurse to attend the nearest health facility for further tests and treatment. Results are not shared with anyone at the school or outside the school without the student’s permission. There is no follow-up of students who are referred. If the student gives permission, then the case is discussed with the LO teacher, who then is expected to follow up.

All the counsellors found it very stressful to give a positive result to a young person and expressed concern that they did not know whether the student actually attended the health facility for follow-up or whether the student had support once the team had left. A counsellor explained …

… it’s really difficult when it’s at the schools because what we’ve found, like, from there some of the children couldn’t handle it and there wasn’t anybody to assist them … they still get referred to the clinic. We don’t get to see them again after that … But if there was somebody that … from there that can assist the child immediately then it’s fine, then you don’t feel so, like … because what does a referral letter do? We can give the child that referral letter. Is she going to go? (Counsellor)

#### Educational materials

Counsellors reported that they did not make use of any visual aids (e.g. flipcharts or posters) as these were not available.

The service providers reported that they had previously handed out pamphlets in English, Afrikaans, and isiXhosa which they fetched from the Department of Health Resource Centre, but they had no stock when the observed site visits were done. The project manager said that the last time he went to fetch stock, he found that the resource centre did not have appropriate materials for schools. What was available was aimed at adults, and were not in line with what was being taught in the curriculum.

#### Student involvement

According to the service providers, besides being tested, except at School B, the students were not involved in the organisation of the HCT process in any way and there was no formal way for students to suggest or recommend changes to the mobile HCT service. At School B, the contact teacher reported that she asked for feedback from the students and then compiled a report of the positive and negative experiences of the testing service which she gave to the service providers. At this school, they also had peer counsellors who helped with co-ordination of the testing days, and the service providers felt that this had been helpful.

### Findings of the observation of the HCT site

Two HCT site visits were done. At the first site visit (School A), the project manager, two nurses, and four counsellors were present, and at the second site visit (School B), the project manager, one nurse (the other was off sick), and four counsellors were present. At both schools, Grade 9 classes were being tested on the day of the visit.

#### General observations

The mobile school-based HCT service did not put any restriction on the use of the service based on race, gender, religion, or socio-economic status. The service was provided free of charge.

#### School A HCT site visit

**Fig. 1.**
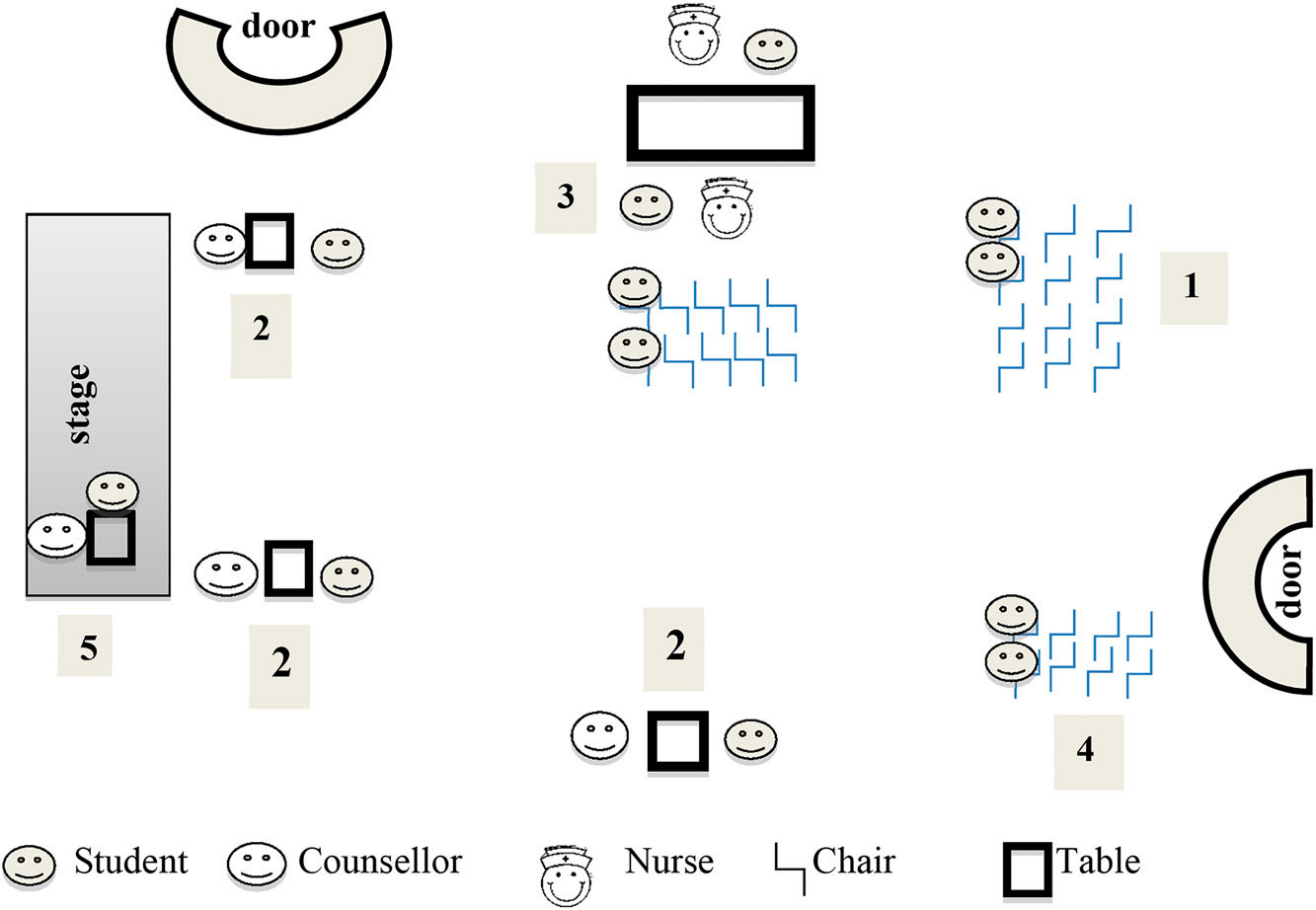
Site layout and environment at school A.

At School A, HCT took place in the hall. The hall was clean and light, and approximately 20 × 25 metres. The entrance door was at the back of the hall, and a stage with curtains closed at the front (Fig. 1).

The hall was divided into a counselling area, a nurses’ station, and three waiting areas (one for pretest counselling, one for the testing station, and one for the post-test counselling). The counselling area was divided into four counselling stations. Three of the stations were set up below the stage at the front of the hall. Each station consisted of a table and two chairs. No screens were set up to provide visual privacy. The stations were about 10 metres apart, providing auditory privacy. The fourth station, where results were given and post-test counselling took place, was behind the stage curtain.

The LO teacher was present for a short whilst to check whether there were any problems. The rest of the time, the project manager made sure that students were well behaved, and directed the flow of students. The students seemed relaxed, and the atmosphere was light. The counsellors and nurses appeared rushed.

#### Process

(1) Students (males and females simultaneously) came down to the testing site, class by class (approximately 30–40 students), according to a schedule drawn up by the LO teacher. They entered at the back of the hall and took a seat at the first group of chairs. The project manager addressed them about the prevalence of HIV, the benefits of testing, and the testing procedure, and stressed confidentiality. The talk lasted for about 10 minutes, and was repeated each time a new class came to the hall. After the talk, those who did not want to be tested then went back to class. (2) Students who wished to be tested were then called up one by one for pretest counselling, which took about five minutes per student. (3) After counselling, they proceeded to the next set of chairs facing the nurses’ station, waiting to be called to have their fingers pricked. No attempt was made at providing visual or auditory privacy. Two students at a time sat on either side of the table with the nurse who was taking their blood. The nurses explained to the students what they were going to do, but did not ask the students any questions. (4) Once blood had been taken, the students went to the post-testing counselling waiting area, where they waited for 15–20 minutes for the nurse to take their results to the counsellor who was doing post-test counselling. (5) Individual students were then called to the counsellor on the stage behind the curtain for their results, and post-test counselling. Post-test counselling took 30 seconds to one minute. No students tested positive during this observation. The process from registration to post-test counselling took approximately 40 minutes per student (the length of one lesson).

#### School B HCT site visit

**Fig. 2.**
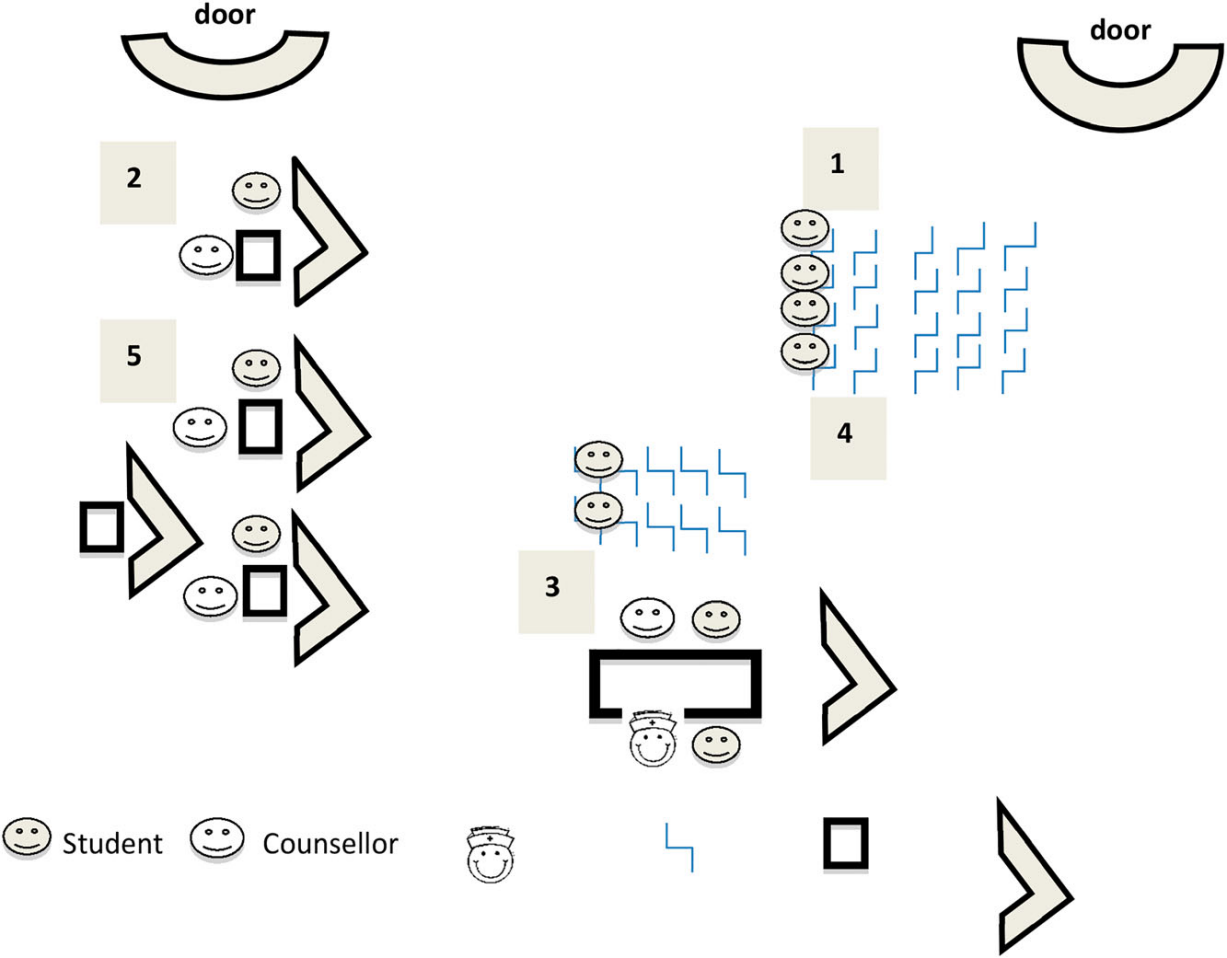
Site layout and environment at school B.

At School B, HCT also took place in the hall, which was approximately 15 × 20 metres. It was divided into a counselling area, a nurses’ station, and two waiting areas. The counselling area was divided into four counselling stations with screens to provide privacy and far enough apart to avoid other students overhearing what was being said. Although students waiting to be seen could not see those behind the screens, students sitting in stations next to each other could see one another. The nurses’ station consisted of a long table with two chairs on either side of it, facing each other (Fig. 2).

The LO teacher was present throughout to ensure that students behaved and to assist the service providers with the flow of students. The atmosphere was light and informal. Most students seemed relaxed, though a few expressed anxiety about having their fingers pricked. The counsellors and nurse appeared rushed.

#### Process

The process at School B was similar to that at School A. Male and female students came down to the testing site class by class, according to a schedule drawn up by the LO teacher. (1) Students entered the hall at the side door furthest away from the counselling stations. After signing in, students took a seat at the first group of chairs facing the counselling stations where, as in School A, the project manager gave them a talk on similar topics. The rest of the process was similar to that at School A: (2) students went for pretest counselling, (3) had blood taken for the HIV test at the nurses’ station (there were no screens here, so students could be seen and heard), (4) waited for 15 minutes for their results, and (5) went for post-test counselling. Again post-test counselling took 30 seconds to one minute. In fact, one counsellor did not even wait for the students to sit down before she told them their results. Here too, the process from registration to the end of post-test counselling took approximately 40 minutes per student (the length of one lesson). It took on average 45–60 minutes to test one class, so the team was able to test about four classes a day.

### Findings of the direct observation of the HCT counselling process

Twenty-one counselling sessions were observed: 14 pretest sessions and 7 post-test sessions (not of the same students observed in the pretest sessions).

#### Quality of counselling

Table 2 shows the counselling skills displayed by counsellors during both the pre- and post-test counselling sessions.

**Table 2.** Number of sessions observed where counsellors adequately demonstrated counselling skills according to the UNAIDS Tool 4 (*n* = 21).

Function	Skills	Counselling session *n* (%)
Interpersonal relationship	Greets clients	21 (100)
	Introduces self	21 (100)
	Engages client in conversation	Difficult to assess
	Listens actively (verbal/non-verbal)	Difficult to assess
	Is supportive and non-judgemental	21 (100)
Gathering information	Uses appropriate balance of open and closed questions	0 (0)
	Uses silence well to allow for self-expression	Difficult to assess
	Seeks clarification about information given	1 (5)
	Avoids premature conclusions	Difficult to assess
	Probes appropriately	9 (43)
	Summarises main issues discussed	0 (0)
Giving information	Gives information in clear and simple terms	16 (76)
	Gives client time to absorb information and to respond	0 (0)
	Has up-to-date knowledge about HIV	Difficult to assess
	Repeats and reinforces important information	0 (0)
	Checks for understanding/misunderstanding	8 (38)
	Summarises main issues	0 (0)
Handling special circumstances	Accommodates language difficulty	21 (100)
	Talks about sensitive issues plainly and appropriately to the culture	21 (100)
	Prioritises issues to cope with limited time in short contacts	21 (100)
	Uses silences well to deal with difficult emotions	Difficult to assess
	Is innovative in overcoming constraints (e.g. space for privacy)	1 (5)
	Manages client’s distress	Difficult to assess
	Flexible in involving partner or significant other	1 (5)

#### Interpersonal relationship

All counsellors greeted the student being counselled and introduced themselves. They were friendly, respectful, and non-judgemental. Because sessions were short and rushed, they did not engage students in conversation, and it was difficult to assess whether counsellors were listening actively.

#### Gathering information

Counsellors asked closed questions and did not probe or seek clarification about the information given.

#### Giving information

Because the sessions observed were with Grade 9 students, the counsellors assumed that the students had learned about HIV at school or when they were previously tested, so little information was given. When information was given, however, it was not always given in clear and simple terms. For instance, a word like ‘contraception’ was used (the student asked for clarification), and the acronym ‘STI’ and the term ‘safe sex’ were used; the student did not ask for clarification, but later the counsellor realised that the term had not been understood. The counsellors did not check whether the information given was understood.

#### Handling special circumstances

All of the students observed were fluent in the language of counselling. The counsellors seemed comfortable to talk about sensitive issues (e.g. sores on the penis). None of the observed sessions elicited obviously upsetting emotions or distress in the students, so the counsellors’ skill in dealing with these situations could not be assessed. Only one student was asked whether his partner had been tested. With regard to sexual orientation, the counsellors assumed that students were heterosexual and posed questions such as ‘Do you have a boyfriend?’ and ‘Do you use contraception?’ to the females.

#### Content of pretest counselling

Table 3 shows the content covered by the counsellors during pretest counselling.

**Table 3.** Assessment of content of pretest counselling (*n* = 14).

During the session have the following occurred?	Counselling sessions in which content aspect/s were adequately covered *n* (%)
Reason for attending discussed	14 (100)
Knowledge about HIV and modes of transmission explored	9 (64)
Misconceptions corrected	0 (0)
Assessment of personal risk profile carried out	9 (64)
Information concerning the HIV test given (e.g. process of testing, window period)	1 (7)
Understanding checked for	14 (100)
Discussion of meaning of HIV-positive and HIV-negative results and possible implications	0 (0)
Capacity to cope with HIV-positive result	0 (0)
Discussion of potential needs and available support	0 (0)
Discussion of a personal risk-reduction plan	0 (0)
Time allowed to think through issues	0 (0)
Informed consent/dissent given freely	14 (100)
Follow-up arrangements discussed	0 (0)
Adequate time for questions and clarifications	0 (0)

During the pretest counselling session, reasons for attending were discussed. Knowledge about HIV and modes of transmission was not really explored, and information concerning the HIV test was not given (e.g. process of testing, meaning of possible test results, and window period). A personal risk profile was carried out by asking students whether they had had unsafe sex and whether they had had contact with blood. They were also asked questions to screen for Tuberculosis (TB) and STIs.

The meaning of an HIV-positive or an HIV-negative result and possible implications, and the student’s capacity to cope with an HIV-positive result were not discussed. The student’s potential needs and available support, should he/she test positive, were similarly not discussed. A personal risk-reduction plan was not explored.

#### Content of post-test counselling

Only post-test counselling sessions in which a negative HIV result was given were observed. No students tested positive in the time period that the sessions were observed. Table 4 shows the content covered by the counsellors during post-test counselling.

**Table 4.** Assessment of content of post-test counselling (*n* = 7).

During the session have the following occurred?	Counselling sessions in which content aspects were adequately covered *n* (%)
Results given simply and clearly	7 (100)
Time allowed for the result to sink in	0 (0)
Checking for understanding	7 (100)
Discussion of the meaning of the result for the client	7 (100)
Discussion of the personal, family, and social implications including who, if any, to tell	0 (0)
Discussion of a personal risk-reduction plan	0 (0)
Dealing with immediate emotional reactions	0 (0)
Checking the availability of adequate immediate support	Not applicable
Discussion of follow-up care and support	Not applicable
Options and resources identified	Not applicable
Immediate plans, intentions, and actions reviewed	Not applicable
Follow-up plans discussed and referrals where necessary	Not applicable

The counsellors gave the results by telling the students that they ‘tested HIV negative’. They *did* ask the students whether they understood the results, but did not clarify this by asking them to explain what they had understood. The meaning and implications of the result for the student were mentioned but not really explored:

Counsellor: And how do you feel about your results?

Student: Happy.

Counsellor: Happy, happy and I’m also happy for you.

The counsellor encouraged students to stay negative, but a personal risk-reduction plan was not discussed:

Counsellor: And just keep it negative, né? You know how to keep your status negative, né? By maintaining a good healthy, positive lifestyle. Is that okay with you?

Student: Okay.

Counsellor: You can go now. Enjoy the rest of your day and the weekend.

Because all results were negative, discussions regarding immediate support, options, and referrals were not needed. Once again, the post-test counselling sessions were very brief

## Discussion

The aim of this study was to evaluate whether the mobile school-based HCT service was youth friendly; that is, did it provide a service which was ‘equitable, accessible, acceptable, appropriate and effective for youth’? (WHO 2002).

### Equitability of the service

The WHO (2012) advocates that policies and procedures should not restrict the provision of health services based on characteristics such as gender, race, and religion and that healthcare providers (as well as support staff) should treat all adolescent clients with equal care and respect, regardless of their status. The counselling provided by the HCT service providers did not take into consideration the special needs of marginalised groups such as young men who have sex with men (MSM), and young people involved in transactional sex and intergenerational sex. Recent studies suggest that South Africa may be experiencing a parallel homosexual and heterosexual HIV epidemic, with 9.8% of 15- to 19-year-old MSM and 49% of 20- to 24-year-old MSM being HIV positive, compared to 3.3% and 6% in the same age groups in the general population (Metcalf & Rispel 2009). A national survey indicated there was a substantive increase in the percentage of youth (specifically young females) who reported having had a sexual partner who was more than five years their senior. Therefore, service providers need to be aware that some students may belong to these marginalised (Shisana, Rehle, Simbayi, Zuma, Jooste, Pillay-vanWyk, *et al*. 2009) high-risk groups, and tailor their counselling appropriately.

The environment in which the HCT service is provided is not the same at all schools, and is dependent on the resources of the school. For example, School B has the resources to provide cubicles so that testing can take place with some visual and auditory privacy, whereas at School A, a poorly resourced school, testing takes place at tables, without any visual privacy. The service needs to be provided with the same high-quality standards at all schools.

### Accessibility of the service

The WHO (2002) defines *accessibility* as meaning that policies and procedures should ensure that health services are either free or affordable to adolescents; that point-of-service delivery should have convenient working hours; that adolescents should be well informed about the range of health services available and how to obtain them; that community members should understand the benefits that adolescents will gain by obtaining the health services they need, and support their provision; and that some health services should be provided to adolescents in the community.

The school-based HCT service is provided free of charge, and by bringing the service to the school, all students at the schools involved are given easy access to the service. The service is provided at a time which is convenient for both the students and the school, and every effort is made to keep disruption to teaching time to a minimum. Nonetheless, some of the students were only informed about the service a few minutes before testing, and educational materials were not given, so they were not well informed to make decisions about using the service.

### Acceptability of the service

An acceptable health service is one which takes into account the preferences and wishes of clients and the cultures of their communities (WHO 2006). The WHO (2012) advocated that a youth-friendly health service (YFHS) should have policies and procedures in place that guarantee client confidentiality; that the point of service delivery should ensure privacy; that short waiting times and swift referral be ensured; that providers should be non-judgmental, considerate, and easy to relate to; that the point-of-service delivery should be appealing and clean; and that information and education should be provided through a variety of channels. They also recommended that adolescents be actively involved in the provision of these health services, to ensure acceptability of the service (WHO 2012). Adolescent focus group participants in a study done in South Africa likewise felt that the assurance of confidentiality and privacy, as well as education about the benefits of testing, made HCT more acceptable (Ntsepe, Simbayi, Shisana, Rehle, Mabaso, Ncitakalo *et al*. 2014).

Concerning the role the client–provider interaction plays in making the service satisfactory, the service providers were friendly and non-judgemental towards the students. With regard to confidentiality and privacy, the NGO ensures confidentiality, but the arrangement of the HCT sites neither provides sufficient auditory nor visual privacy. The HCT sites were clean, but no attempts were made by the service providers or school to make the environment appealing to young people, for instance, putting up posters, playing music, or providing games to play (all possible with limited resources).

The WHO (2012) advised that in order to increase the likelihood that a service is found acceptable to young people, youth clients should be involved in designing, assessing, and providing the service. According to the project manager, he had not considered including students in the provision of the service as he thought that they did not have time to be involved. School B, however, had taken the initiative to involve their peer counsellors to assist with the smooth running of the testing day, and they gave students the opportunity to give feedback about the service, and then acted upon the feedback.

### Appropriateness of the service

The WHO (2012) defines an appropriate service for young people as one which provides the required package of health care that fulfils the needs of all adolescents, either at the point of service or by referral to the necessary facility. HCT is a service which has been advocated both internationally (UNAIDS 1997) and locally (South African National AIDS Council 2007) as an appropriate way of effectively preventing the transmission of HIV by providing a critical entry point for care and treatment for those who test positive (Janssen, Holtgrave, Valdiserri, Shepherd, Gayle & De Cock 2001; Mshana, Wamoyi, Busza, Zaba, Changalucha, Kaluvya, *et al*. 2006; Nsigaye, Wringe, Roura, Kalluvya, Urassa, Busza, *et al*. 2009; Perbost, Malafronte, Pradier, Santo, Dunais, Counillon, *et al*. 2005). Authors of other studies have similarly intimated that HCT is appropriate and consistent with the preferences of young people (UNAIDS 2001).

However, in this case, HCT is not provided as part of a comprehensive school-based health service (which includes reproductive health services, TB screening, nutrition services, mental health services, and social services). If it was, students would be able to visit the service provider without anyone else knowing their reason for the visit. Also, providing HCT as part of a package of health services has the potential to normalise it.

### Effectiveness of the service

With regard to YFHS, the WHO (2012) recommended that in order to offer an effective health service to young people, healthcare providers should have the required competencies to work with adolescents and to provide them with the required health services; that evidence-based protocols and guidelines should be used to provide health services; that healthcare providers should be able to dedicate sufficient time to dealing effectively with their adolescent clients; and that the point-of-service delivery should have the required equipment, supplies, and basic services necessary to deliver the required health services.

The service providers had minimal experience of working with young people and had not received any training in dealing with young clients. Also, the mobile service did not have sufficient human resources and equipment. The counselling sessions were too short and rushed to build up any sort of rapport between the student and the provider, and there was little room for the student to have a voice. One of the counsellors expressed her unhappiness with having to rush through counselling clients, because counsellors were expected to see 20 students in a day. In order to provide a quality service that meets the needs of the students, there is a need for sufficient counsellors to ensure that student–provider interaction is long enough to provide counselling which is of value to the student. The service providers did not have the necessary equipment (tents or cubicles) to provide auditory and visual privacy during counselling. They did not have educational materials (pamphlets and posters), or provide extensive publicity so that students were well enough informed to make decisions about using the service.

Furthermore, the service providers raised concerns that although students who tested positive were referred to a health facility for further care and treatment, the service providers did not provide follow-up, so they were unsure whether these students actually received the care that they needed. This concern about poor linkage to care after testing HIV positive has been raised by other authors (Bekker, Johnson, Wallace & Hosek 2015; Kurth, Lally, Choko, Inwani & Fortenberry 2015; Tanner, Philbin, Duval, Ellen, Kapogiannis & Fortenberry 2014).

## Limitations

A limitation of this study is that the evaluation was done by a single individual and not a team, which may have resulted in interviewer/observer bias. However, the findings across the three methods of data collection (observation of HCT sites, interviews of service providers, and direct observation of HCT counselling sessions) corroborated each other and no inconsistencies were noted. Because of the small sample size and the fact that a purposeful sample was selected, data from this study cannot readily be generalised.

## Conclusions and recommendations

Providing HCT in the school setting may make HCT more accessible for school-going young people, but should be provided in an equitable, acceptable, and effective way. Based on the findings of this evaluation, service providers should receive training (and then be monitored and supported) to work with young people, which should include the special needs of marginalised groups. The model of ‘mass testing’ described in this study (where students are brought to the HCT site in groups) is not acceptable for young people and does not fulfil their expressed need for privacy with regard to HCT. Mechanisms need to be developed to ensure that students are able to test without being seen, and this should apply to all schools, irrespective of the resources available to them. Students who test positive should be linked to the necessary care and support. Referral networks should be set up, and young people need to be referred to specific individuals within HIV services who have training and experience in working with young people. If the emphasis of school-based HCT is on increasing the number of students who consent to being tested in order to identify students who are positive (as opposed to counselling focused on changing risky behaviour), then students should be given another space to discuss issues that would normally be addressed in post-test counselling, such as prevention plans.

Further studies are needed to explore why students who test positive do not receive the care that they need. Other school-based HCT services should be evaluated to identify best practices and to develop a model for providing youth-friendly HCT in the school setting.

## Notes

1. NGO project manager, personal communication, 2010.

2. The terms *adolescent-friendly* and *youth-friendly* health services are used interchangeably in the literature. In this study, the term *youth friendly* is used as the participants include young people up to the age of 24 years. The WHO defines ‘adolescence’ as 10–19 years of age, ‘youth’ as 15–24 years of age, and ‘young people’ as 10–24 years of age (WHO 1986).

3. Life Orientation is
the study of the self in relation to others and to society. It addresses skills, knowledge, and values about the self, the environment, responsible citizenship, a healthy and productive life, social engagement, recreation and physical activity, careers and career choices. (South African Department of Basic Education 2011:7)

4. ACTS (Advise, Consent, Test, Support) is a system aimed at making HCT easier for healthcare providers to integrate into the other services they routinely deliver by reducing the conventional UNAIDS 25-minute pretest counselling session to 5 minutes or less. It is also meant to allow lay counsellors to test more clients per day and frees up their time to provide more intensive counselling to HIV-positive clients. ACTS was developed in the USA and has been implemented by the Western Cape Department of Health since 2009. See Appendix 3 for steps followed during the counselling process.

## Appendix 1. Amendments made to ‘Clinic assessment of youth friendly services tool’

The terms ‘client’ and ‘youth’ were changed to ‘learner’ throughout the document, as that is the term used by the South African Department of Basic Education.

Section II: ‘Range of services provided’ and ‘Schedule of available services’ (sections removed)

Section IV:

1. Location: Question added: *Where at the school is the service provided?*
  Questions removed: *How far is the facility from public transportation? How far is the facility from places where adolescents spend free time? How far is the facility from schools in the area?*
2. Facility hours: The heading ‘Facility hours’ was changed to ‘HCT service hours’.
  *What time is the clinic scheduled to open?* was changed to *What time is the service provided?*
  Questions removed: *What time is the clinic scheduled to close? Does the facility have separate hours for adolescents? Is there a sign listing services and clinic working hours?*
3. Facility environment: The word ‘facility’ was changed to ‘site’.
  Questions removed: *Does the facility have a separate space to provide services for adolescent clients? Does the facility have a separate waiting room for adolescent clients? Is there an examination room that provides visual and auditory privacy?*
4. Services provided: Questions removed: *What contraceptive methods are offered? Is there sufficient equipment for the provision of reproductive health services for young people?*
5. Peer education/counselling programme (section removed).
6. Support policies: Questions removed: *Are there any contraceptive methods that adolescents cannot receive? Are adolescent clients served without regard to their marital status? Are pelvic exams routinely required?*
7. Administrative procedures: Question removed: *If appointments are required, can they be expedited for adolescent clients?*

## Appendix 3. The ACTS model.

Retrieved from: http://www.actshivtest.org.za/ACTS%20A3.pdf
